# Extraosseous marrow fat: an MRI sign of acute aggressive osteomyelitis

**DOI:** 10.1259/bjrcr.20180050

**Published:** 2018-09-28

**Authors:** Dorissa Lahner Gursahaney, MK Jesse, Jason Stoneback

**Affiliations:** 1 Department of Radiology, School of Medicine, University of Colorado, Aurora, CO, USA; 2 Department of Orthopedics, School of Medicine, University of Colorado, Aurora, CO, USA

## Abstract

MRI plays a critical role in the evaluation of osteomyelitis. However, MRI findings of osteomyelitis are not entirely specific and may mimic infiltrative tumors. We describe a case of extraosseous extruded medullary fat with a tiny transcortical tract caused by acute osteomyelitis, diagnosed by MRI and confirmed with intraoperative findings and pathology. Identification of extruded medullary fat is a specific MRI finding that aids in the differentiation of acute osteomyelitis from infiltrative tumor thereby preventing unnecessary biopsy and facilitating prompt diagnosis and initiation of appropriate treatment.

## Case presentation

A 62-year-old male with a history of diabetes mellitus and rheumatoid arthritis, treated with leflunomide and rituximab, presented with bacteremia and acute onset right knee pain. Physical examination revealed right knee and proximal tibia tenderness and erythema. Initial laboratory work-up revealed gram-positive cocci bacteremia, elevated C-reactive protein, and right knee joint aspiration with no organisms seen on gram stain.

## Investigations

**Figure 1.**
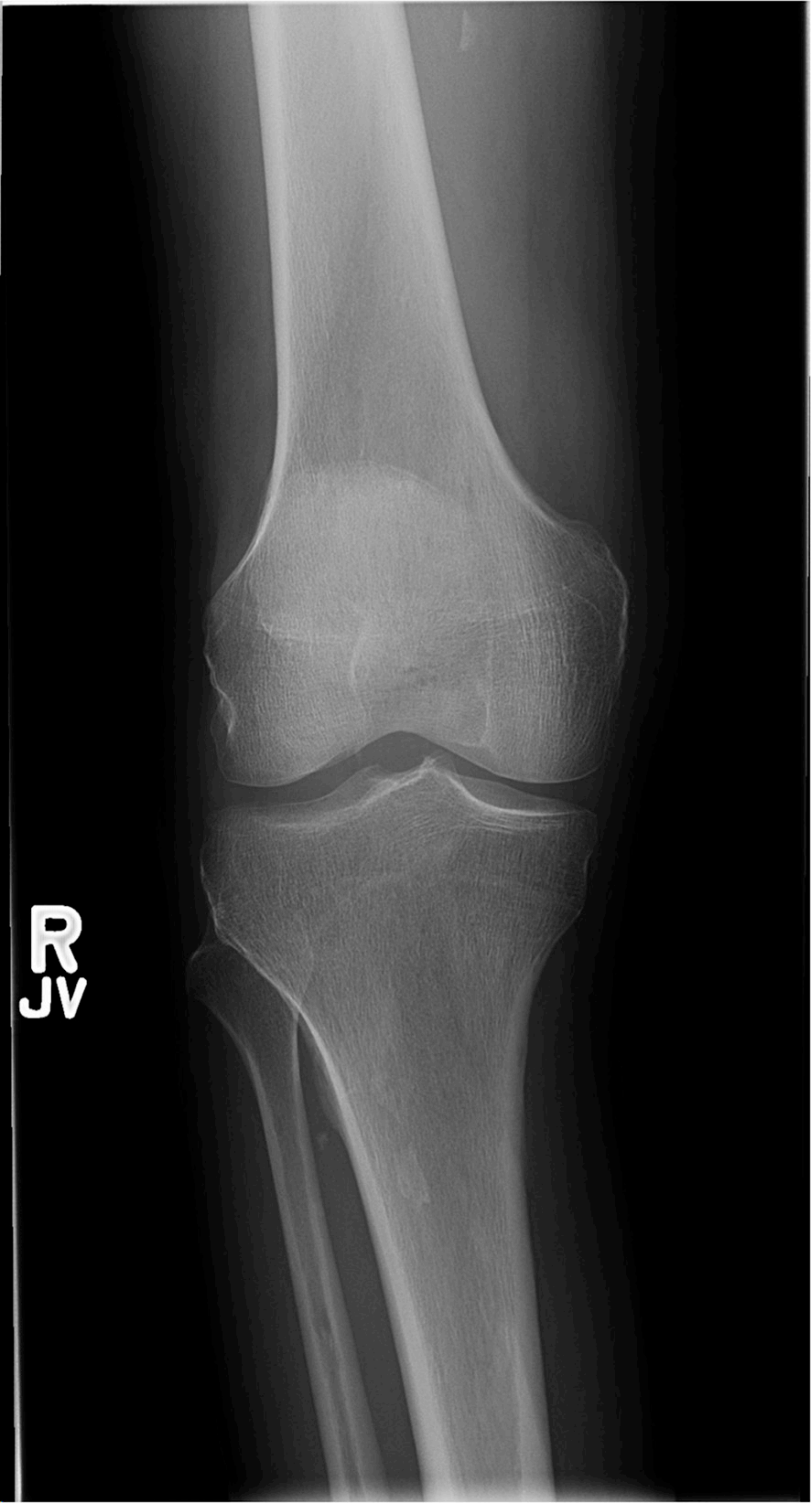
A 62-year-old male with history of rheumatoid arthritis presents with acute right knee and proximal tibial pain. Normal right knee radiograph.

**Figure 2.**
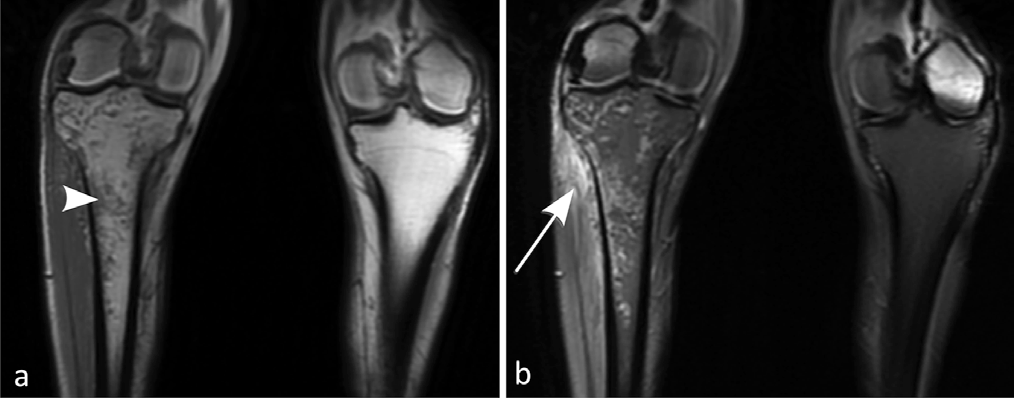
(a-b) Coronal MRI of the tibia and fibula. *T*_1_ weighted image (a) reveals heterogeneous marrow signal (arrowhead) within the proximal tibia with wispy confluence of T_1_ signal. *T*_2_ weighted image (b) demonstrates linear signal and periostitis along the lateral tibial shaft (arrow) with intermittent reactive marrow signal.

**Figure 3.**
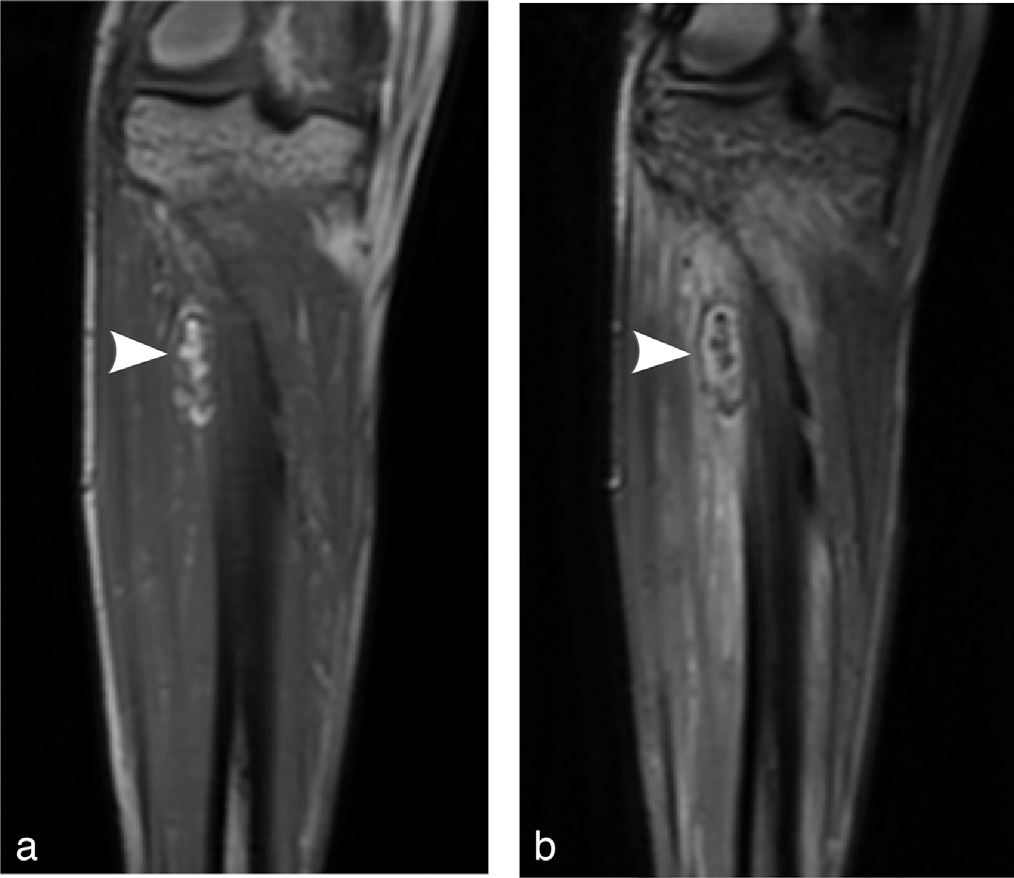
(a-b). Coronal MRI of the tibia and fibula. *T*_1_ weighted (a) sequence reveals a small foci of T_1_ signal along the outer cortex of the proximal tibial shaft (arrowhead) and *T*_2_ weighted fat saturated sequence (b) demonstrating clear fat saturation (arrowhead) compatible with macroscopic fat.

**Figure 4.**
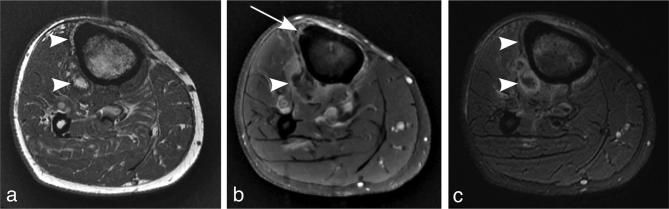
(a-c) Axial *T*_1_ weighted non-contrast (a), *T*_1_ weighted post-contrast fat saturation (b), and T_2_ fat saturation (c) MRI of the tibia and fibula at the level of the proximal tibial metaphysis. Single slice finding of a tiny sinus tract through anterior tibial cortex (arrow) with macroscopic fat tracking to the larger fat containing extraosseous collection (arrowheads).

Imaging of the right knee, tibia, and fibula was preformed to evaluate for a source of pain. Radiographs were normal (Figure 1). MRI with and without contrast of the right tibia and fibula, the site of greatest clinical suspicion, revealed diffuse heterogeneous marrow edema throughout the proximal tibial metadiaphysis with extensive periosteal edema compatible with an infiltrative process. Further evaluation on T_1_ imaging revealed small foci of extraosseous macroscopic fat tracking from a tiny cortical defect in the anterior tibial cortex (Figures 2–4).

## Differential diagnosis

Given the diffuse though well-defined heterogeneous signal abnormalities asymmetric within the tibial shaft, initial diagnostic consideration included osteomyelitis *v* *s* infiltrative or hematological malignancy *vs* systemic marrow process. The identification of a transcortical tract with expulsion of marrow fat to the extraosseous soft tissues, however, is considered a highly specific finding in acute osteomyelitis effectively narrowing the differential to a single diagnosis and preventing an unnecessary biopsy to exclude malignancy.

## Treatment

**Figure 5.**
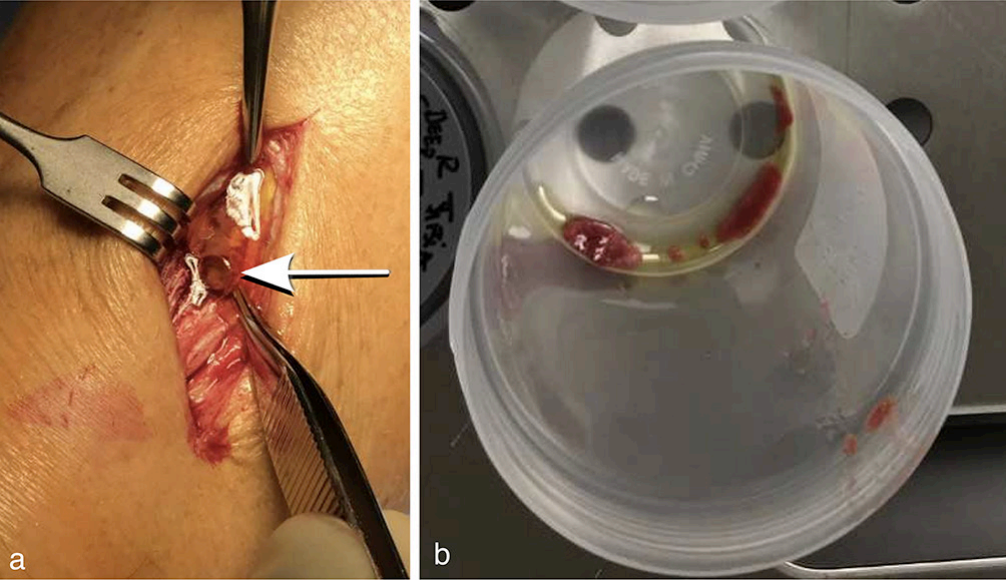
(a-b). Intraoperative findings overlying the proximal tibia (a) demonstrate extruded extraosseous fat (arrow) and surgical specimen (b) with macroscopic extraosseous fat.

Empiric vancomycin was subsequently narrowed to cefazolin following blood culture sensitivities revealing methicillin-sensitive Staphylococcus aureus. With the definitive imaging diagnosis of acute osteomyelitis, continued positive blood cultures, and progressive symptomatology despite appropriate antibiotic treatment, the patient was referred to The Limb Restoration Program for multidisciplinary evaluation. The multidisciplinary program group decision was for urgent surgical irrigation, debridement, and saucerization of the proximal tibial metadiaphysis as the patient’s immunocompromised state limited his ability to clear the infection with systemic therapy alone. Intraoperative findings were notable for macroscopic extraosseous fat and an adjacent osteomyelitic tibial sinus track (Figure 5). Deep soft tissue biopsy of this area was sent for permanent pathological analysis, culture, and sensitivity. Biopsy analysis revealed histological findings consistent with acute osteomyelitis and microbiology findings consistent with MSSA.

## Outcome and follow-up

Prior to hospital discharge, the patient’s blood cultures revealed no growth and a peripherally inserted central catheter was placed to complete a 6-week course of cefazolin. Upon outpatient follow-up after completing 2 weeks of cefazolin, the patient was found to have a healing wound site and no systemic signs or symptoms of infection.

## Discussion

Osteomyelitis, infectious inflammation of the bone marrow, is a cause of significant morbidity and mortality. Although MRI is the most sensitive imaging modality for diagnosing osteomyelitis, imaging features can be confusing often mimicking aggressive infiltrative tumor. Overlapping clinical features of osteomyelitis and skeletal neoplasm, including focal pain, fever, and elevated inflammatory markers, further complicate this diagnostic dilemma. Osteomyelitis has been previously described masquerading as Ewing sarcoma, chondrosarcoma, and other skeletal neoplasms.^1,2^ Radiographic misdiagnosis of osteomyelitis is especially notable in the pediatric population where it is estimated that half of subacute osteomyelitis cases are misdiagnosed as tumor.^3^ The imaging feature of extraosseous marrow fat in the region of marrow signal abnormality is a highly specific finding in acute and aggressive osteomyelitis and may assist in the differentiating of these entities by imaging alone.^4^

Understanding of the pathophysiology of osteomyelitis facilitates the interpretation of extraosseous fat signal when there is clinical concern for osteomyelitis. Acute superative response from medullary bacterial proliferation can cause an acute increase in intramedullary pressure, ultimately leading to a rupture of the cortex and expulsion of medullary fat into the adjacent soft tissues.^4,5^ Interestingly, foci of extraosseous fat are predominately a feature of acute osteomyelitis and not identified in subacute osteomyelitis patients.^4^ This observation is in keeping with the pathophysiology of aggressive acute osteomyelitis resulting in extrusion of medullary fat as opposed to a subacute, slowly progressive process.

There are few publications documenting the imaging finding of extraosseous fat as a specific feature of acute osteomyelitis.^4,6,7^ To our knowledge, this is the only reported case confirmed with both bone biopsy and intraoperative findings documenting a cortical sinus tract with macroscopic extraosseous fat. Extraosseous fat fluid levels have been elegantly depicted on fat-suppression MRI sequences and previously described as a specific sign for acute osteomyelitis in patients presenting with a soft tissue infection.^6,7^ In the absence of trauma, the extraosseous fat fluid level on MRI has been proposed as a specific sign for osteomyelitis with cortical breach even when the cortical breech was not seen.^7^ In contrast to acute osteomyelitis, the penumbra sign consisting of hyperintense signal interposed between low signal abscess cavity and marrow has been previously described as highly specific for chronic, subacute, or acute on chronic osteomyelitis.^8^

## Learning points

1. Extraosseous marrow fat is a specific imaging feature of acute aggressive osteomyelitis assisting in the differentiation between osteomyelitis and infiltrative tumor, thereby preventing unnecessary biopsy for diagnosis and facilitating prompt treatment.
2. In the absence of trauma, extraosseous marrow fat in the setting of soft tissue infection is indicative of acute osteomyelitis. This may be particularly helpful when the cortical defect is not seen due to wide interslice gaps.
3. Extraosseous medullary fat aids in determining aggressiveness of acute osteomyelitis, as it requires extensive, rapid marrow necrosis in order for the macroscopic fat to accumulate in extraosseous soft tissues.
